# Remote Sensor System for Assessing the Toxicity of Car Exhaust Gases

**DOI:** 10.3390/s26061928

**Published:** 2026-03-19

**Authors:** Krzysztof Więcławski, Jędrzej Mączak, Krzysztof Szczurowski

**Affiliations:** Faculty of Automotive and Construction Machinery Engineering, Warsaw University of Technology, 05-524 Warsaw, Poland; jedrzej.maczak@pw.edu.pl (J.M.); krzysztof.szczurowski@pw.edu.pl (K.S.)

**Keywords:** sensors, vehicle exhaust, mobile measurement

## Abstract

This paper presents the design of a sensor system for remote measurements of exhaust emissions from automotive combustion engines. The system’s purpose is to determine the likelihood of a given vehicle’s potential harmfulness to the environment. This system, if implemented, could detect vehicles posing a threat to the environment in road traffic. A remote measurement system can be installed in the front of a measuring vehicle driving behind the vehicle being diagnosed. This approach allows for rapid road testing of multiple vehicles while they are operating in real-world conditions where engines can emit the highest levels of undesirable pollutants. Exceeding emission standards may be related to modifications made to the vehicle’s exhaust gas aftertreatment systems, engine wear, or malfunctions of engine-related systems such as the diesel particulate filter (DPF) or catalytic converter. Toxic and undesirable substances include carbon monoxide (CO), hydrocarbons (HC), nitrogen oxides (NO*_x_*), carbon dioxide (${CO}_{2}$), and particulate matter (PM) particles. The main goal of the measurements is to identify vehicles that potentially pose a threat to the environment during normal operation. The sensor system consists of several types of sensors utilizing various physical and chemical phenomena, with particular emphasis on their low cost and easy availability. The measurement unit utilizes MEMS technology, photoacoustic spectroscopy, electrochemical methods, light absorption and scattering, spectrophotometry, and electro-optical detection.

## 1. Introduction

Exceeding the emission standards may be related to modifications made to the vehicle exhaust gas purification systems, engine wear or damage to engine-related systems such as the diesel particulate filter (DPF) or catalytic converter [1]. Environmental pollution from internal combustion engines is a constant problem, especially in areas with high vehicle traffic density [2,3]. This is especially true when vehicles in traffic do not meet the required exhaust emission standards due to wear, modifications, or damage. Toxic and undesirable substances include carbon monoxide (CO), hydrocarbons (HC), nitrogen oxides (NO*_x_*), and carbon dioxide (${CO}_{2}$) [4,5,6]. Detection of a given compound in the air is possible due to the individual physical and chemical properties of the tested gases detected by the sensors. Components of such a system are subject to rapid degradation due to the aggressive effects of smoke particles and therefore require frequent replacement.

Exhaust gas mobile measurement is a challenging issue because vehicles generate the most toxic substances while driving, which can lead to relatively rapid system contamination. Regenerative systems can be used, but this increases the cost and complexity of the system and reduces its efficiency. Hence, easily replaceable components are used. Mobile systems described in the subject literature mostly involve systems built as road gates through which vehicles pass, or systems for testing vehicle emissions that have a measurement system mounted on their own exhaust pipe (PEMS) [7,8,9,10]. Well-known mobile laboratories are sets of complete measurement devices, in the form of a measuring platform towed behind the vehicle [11,12]. These are very efficient and expensive devices, consisting of quite complex and extensive systems, which, however, require intervention in the vehicle [13,14]. J. Rajagukguk et al. [15] describe a system composed of ready-made sensors in an Android environment, used for measurements while driving but located at the exhaust pipe of the tested vehicle. Attention is paid to the influence of external conditions on the measurements, as well as the influence of other vehicles whose exhaust gases may distort the measurements [16]. These mobile measurements were verified in comparison to tests conducted with the use of vehicle dynamometers [17].

The systems discussed in the publications mentioned above are based on measurements taken directly in the vehicle’s exhaust pipe while the vehicle is in motion. The results obtained from these measurements are consistent with stationary measurements. It is clear that these studies accurately reflect the composition of the vehicle’s exhaust gases. The article by Mazzoleni et al. [18] presents a roadside exhaust gas measurement system in the form of a sensor gate mounted across the road. Exhaust gases from passing vehicles are scanned by the wave sources of the measurement systems used, using UV Lidar. The measurements allow for determination of PM particles and ${\mathrm{CO}}_{2}$ level. An analysis of the correlation between the abundance of these particles and CO and HC emissions was also conducted, demonstrating the absence of such a relationship. The authors demonstrated the usefulness of the presented solution in estimating exhaust gas composition in terms of PM abundance and high measurement sensitivity. The discussed system does not use a measuring probe placed in the vehicle’s exhaust pipe, which makes it similar to the one presented by the authors in this article. TPI Sp. z o. o. offers a mobile device called Sniffer4D [19] for measuring air pollution in large areas. The device measures ${\mathrm{CO}}_{2}$, PM_2.5_, PM_10_, sulfur dioxide, and hydrogen sulfide. The company proposes a mobile solution, in which the Sniffer4D is mounted under a drone. This module can be used to test vehicle exhaust gases, but it does not measure CO content, and the duration of its most accurate performance is not specified, which is important due to the potential rapid degradation of measuring devices caused by smoke.

The paper in [20] presents the concept of mobile measurements using an independent ${\mathrm{CO}}_{2}$ sensor that works with a smartphone app. The ${\mathrm{CO}}_{2}$ sensor does not work with any probe. The system is based on the MQ-135 electrochemical sensor. Paper [15] describes an inexpensive and compact sensor system for mobile measurements of the exhaust gas. Optical absorption in the UV range was used with LED-based spectroscopy. ${\mathrm{NO}}_{2}$, ${\mathrm{SO}}_{2}$, and PM levels up to 1000 ppm were detected. The system was designed for use in stationary equipment to test smoke in industrial areas. Measurements were performed using a drone with a mounted measurement kit. Industrial facilities (e.g., port engines) generate incomparably more exhaust gases than automotive (passenger) vehicles, so high measurement efficiency was also possible due to the stationary nature of the facility and the possibility of performing measurements above the facility.

This paper presents a mobile measurement system for remote measurements of exhaust toxicity generated by automotive combustion engines [21]. Unlike the systems described above, this particular system was developed as an experimental laboratory unit enabling research on the selection of appropriate sensors designed to assess the amount of exhaust gases emitted by vehicles in motion, with the aim of selecting the most effective measurement method. It enables the detection of exhaust gases through the analysis of data obtained from a set of sensors without installing measurement hardware on the analyzed vehicle. The aim of this work was to determine whether remote assessment of exhaust pollution levels is possible and whether the results obtained allow for the “positive” and “negative” screening of vehicles in regard to their exhaust emissions, i.e., assessing the likelihood of a given vehicle posing a threat to the natural environment.

The remote measurement system, comprising a set of sensors and controllers, is designed to be mounted on the front of the measuring vehicle, allowing for screening tests of multiple vehicles in a short time and under normal road conditions. For technical reasons, this is an indicative measurement, hence the statement about the probability of a vehicle significantly exceeding the permissible exhaust emission standards. This approach makes quick conducting of many road tests possible, while vehicles emit the most toxic substances. The detection system is composed of various sensors using different physical and chemical phenomena [18,22,23]. The goal was to build a system that would be miniaturized [24], inexpensive, and relatively simple to implement. This system, if implemented, could detect vehicles posing a threat to the environment in road traffic. The system consists of two spectrometers that receive visible, ultraviolet, and infrared radiation generated by a specially selected array of light-emitting elements, which serve as the light source for the spectrometers. This array consists of RGB LEDs, laser diodes and ultraviolet and infrared emitters, with their wavelengths matched to the frequencies of radiation absorbed and scattered by exhaust gases. When exhaust gases are present in the optical radiation path, changes in brightness (attenuation) at specific wavelengths allow for conclusions to be drawn regarding their toxicity. The sensor set includes both commercially available sensors and proprietary solutions. Measurement takes place approximately 2–3 s after the exhaust gases are collected by the fan. The system is managed by dedicated software and employs multiparametric analysis. The acquired data is analyzed and compared with a reference pattern, enabling the assessment of the toxicity level of the measured exhaust gases.

**Figure 1:**
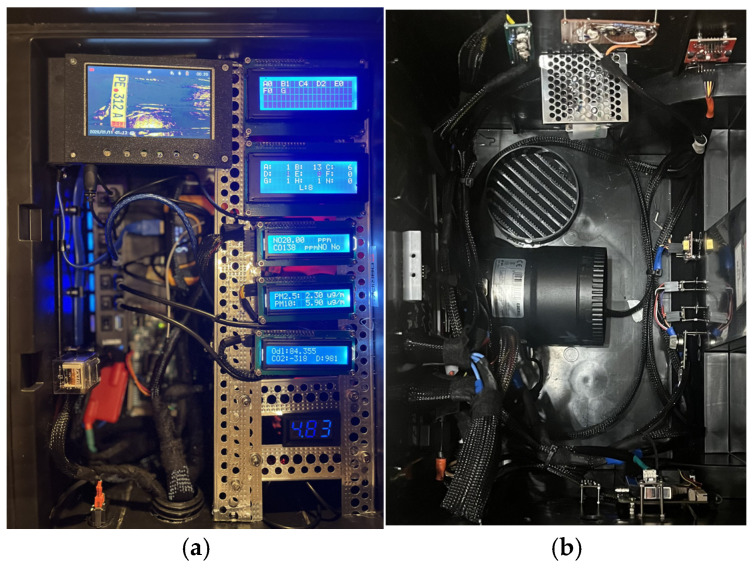
Exhaust gas remote measurement module. Control and display of results (**a**) and sensor module (**b**).

The air intake (housing with the sensors) incorporates a fan that captures exhaust gases from the vehicle ahead, and that serves the purpose of increasing the gas concentration in the housing (Figure 1). The figure on the left shows the control and display of results; on the right is the measurement module.

## 2. Physical Basis of Measurements

Methods that can be implemented for identification of gases include the use of optical phenomena [25,26]. In the following chapter the physical basis of measurements allowing for the determination of the concentration of different gases will be discussed.

The carbon dioxide (${CO}_{2}$) molecule has a linear and symmetrical structure; i.e., the carbon atom is placed between two oxygen atoms. This structure results in the absence of an electric dipole moment, so the emitted photon will not excite the molecule’s vibrations. The excitation of the vibrations of the element’s molecule is related to wave numbers and wavelength. As a result, carbon dioxide is transparent in the visible spectrum. Two vibrational bands are possible—the first being an asymmetric stretching band at 4.5 µm and the second at 14.99 µm—meaning that only at these wavelengths is an absorption spectrum generated, allowing the detection of the carbon dioxide molecule. This is a spectroscopic method [27,28,29]. Weaker absorption bands occur in the bandwidths of 2.0 to 2.7 µm and 14 to 16 µm. These absorption bands are infrared, and the amount of absorbed light is proportional to the gas concentration. Absorption spectroscopy is based on the Beer–Lambert law, which relates the absorption of light by a sample to the concentration of the absorbing material:

(1) A=ε∗c∗l

where

*A*—absorbance;
*ε*—molar absorption coefficients (L·mol^−1^·cm^−1^);
*c*—solution concentration (mol·L^−1^);
*l*—the length of the optical path (cm) through which light passes.

The ratio of the light from the source to the light after passing through the component being tested is

(2) $$A={log}_{10}\left(\frac{I_{0}}{I}\right)$$

where

$I_{0}$—incident light intensity.
*I*—intensity of light after passing through the component.

Photons are absorbed only in their entirety, fulfilling the conservation principle of quantum mechanics, which states that in the absorption process, the photon’s energy depends on the light frequency *ν*. This energy is transferred to the particles through which the electromagnetic wave passes:

(3) $$\Delta E=h\nu =h\frac{c}{\lambda }=hc\bar{\nu }$$

where

*h*—Planck’s constant;
*c*—speed of light;
λ—absorbed wavelength;
$\bar{\nu }$—wave number;
ν—vibration frequency.

The fundamental problem of using this method in mobile measurement is generation and capture of mid- and far-infrared waves. The devices needed for such detection do not meet the requirement of readily available components of the discussed system. Ultraviolet and visible light are not absorbed by carbon dioxide. Using these IR wavelengths in ${\mathrm{CO}}_{2}$ identification is difficult and expensive. Infrared radiation with a wavelength of 4.25 µm is emitted by a body heated to approximately 1000 °C, which shows the scale of the difficulty in using this detection method. This availability requirement is met by photoacoustic spectroscopy, described, among others, in [30]. The photoacoustic effect involves the creation of sound waves, for example, in gas molecules, as a result of the absorption of electromagnetic radiation. When gas molecules illuminated by radiation of a specific wavelength absorb light energy [31,32], according to the Beer–Lambert law, the emitted beam has an intensity dependent on the absorption coefficient and the length along the light path:

(4) $$I(\nu )=I_{0}(\nu )\;e^{-\alpha (\nu )L}$$

where

$I_{0}$(*ν*)—initial intensity;
I(ν)—intensity after passing through the sample;
L—length of the optical path;
*α*(*ν*)—absorption coefficient (is a function of wavelength, temperature and pressure).

The intensity changes depend on the modulation frequency (*ω*) of the light wave:

(5) $$I(t)=I_{0}(1+m_{∆}cos\;\omega t)$$

$m_{∆}$ in the equation is a parameter defining the depth of wave modulation, taking values in the range 0≤m≤1. The absorbed power (per unit volume) results from the modulation of the light wave intensity Q(t):

(6) $$Q(t)=\alpha I(t)=\alpha I_{0}(1+mcos\;\omega t)$$

The absorption of power results in an increase in temperature:

(7) $$\Delta T(t)=\frac{Q(t)}{\rho c_{p}}$$

*ρ*—density $CO_{2}$;
$c_{p}$—specific heat at constant pressure.

According to the gas equation of state, a change in temperature implies a change in pressure:

(8) $$p(t)=(\gamma -1)\rho c_{v}\;\Delta T(t)$$

and as a function of absorbed power:

(9) $$p(t)=(\gamma -1)\frac{Q(t)}{v_{s}^{2}}$$

where

$\gamma =\frac{c_{p}}{c_{v}}$—adiabatic exponent (for ${\mathrm{CO}}_{2}$: γ≈1.30);
$v_{s}$—speed of sound in ${\mathrm{CO}}_{2}$ (~268 m/s at 20 °C).

As a result, a strong photo-acoustic signal is obtained in IR, which allows the detection of CO. The value of p(t) is the total sound pressure (DC+AC):

(10) $$p(t)=(\gamma -1)\frac{\alpha I_{0}}{v_{s}^{2}}(1+m_{∆}cos\;\omega t)$$

The component described as DC is the constant factor of the signal (independent of time):

(11) $$p_{\mathrm{DC}}=(\gamma -1)\frac{\alpha I_{0}}{v_{s}^{2}}$$

The AC component is the signal change, which is the measured photo-acoustic signal:

(12) $$p_{\mathrm{AC}}(t)=\left(\gamma -1\right)\frac{\alpha I_{0}m_{\Delta }}{v_{s}^{2}}\cos\omega t$$

**Figure 2:**
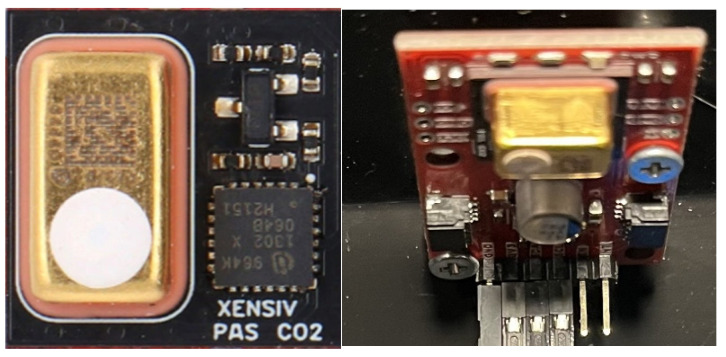
Carbon dioxide XENSIV PASC02V1 sensor.

The $P_{\mathrm{AC}}(t)$ value is the acoustic component responsible for light modulation (amplitude modulated photoacoustic pressure). Due to the energy conversion in the sequence absorption → temperature change → pressure change → acoustic wave, the photoacoustic method detects ${\mathrm{CO}}_{2}$ (Figure 2).

This type of sensor was used in the construction of the measurement module of the discussed system. In this method, an electro-optical detector with a laser light source allows for determination of the ${\mathrm{CO}}_{2}$ concentration in ppm units (parts per million).

PM (particulate matter) particles indicate the level of undesirable particles, whose small size is a problem because they easily penetrate into organisms. PM refers to identified particulate matter, the unit of which is $\mu g/m^{3}$, although the norms of air pollution are using ppm—the number of particles of a substance per million air particles.

For evaluation of the amount of PM particles in the exhaust gases the laser sensor could be used. The equation for the laser light wave at frequency *ν* can be described as

(13) $$E(z,t)=E_{0}cos\;(\omega t-kz+\phi )$$

where

$E_{0}$—amplitude;
*ω*—angular frequency;
$k=\frac{2\pi }{\lambda }$—wave number;
λ—laser wavelength;
ϕ—initial phase.

The interaction of an electromagnetic wave with carbon dioxide is presented in Equation (4). The absorption coefficient *α*(*ν*), after the laser passes through ${\mathrm{CO}}_{2}$ is defined as

(14) $$\alpha (\nu )=S(T)\;g(\nu -\nu_{0},\;p,\;T)\;C$$

where

- S(T)—absorption line strength from HITRAN (a database describing how gas molecules absorb and emit electromagnetic radiation);
- $g(\nu -\nu_{0})$—line profile (Gaussian, Lorentzian or Voigt);
- C—concentration of $CO_{2}$;
- $\nu_{0}$—resonance frequency of a given absorption line.

Gaussian profile is created by Doppler blurring—associated with the thermal motion of molecules. The Lorentzian profile is the result of collisions between molecules (pressure broadening). The Voigt profile combines Gaussian and Lorentzian profiles: Doppler blurring (Gauss) and pressure blurring (Lorentz). In summary, the equation for the laser wave and $CO_{2}$ absorption is as follows:

(15) $$E(z,t)=E_{0}\;e^{-\frac{1}{2}\alpha (\nu )z}cos\;(\omega t-kz+\phi )$$

The equation describes the attenuation of the amplitude of an electromagnetic wave during propagation in a medium. The greater *α*(*ν*), the faster wave decays in the medium. However, the amounts of PM are not identified by absorption, but by scattering of light. In reality, both methods are combined, allowing for determination of the total attenuation as the sum of

- Gas absorption;
- PM attenuation.

Identification of suspended particulate matter (PM) requires a scattering method. PM particles (${\mathrm{PM}}_{1.0}$, ${PM}_{2.5}$, ${PM}_{10}$) range in size from 0.1 to 10 µm, are irregularly shaped, and do not generate narrow spectral lines. These properties are the reason why the detection is based on using Mie/Rayleigh scattering:

(16) $$I_{scattered}=\alpha *\;n_{PM}*\sigma_{scat}(\lambda )$$

where

$I_{\mathrm{scattered}}$—the intensity of the scattered light, measured by a photo detector (the signal is analyzed by a PM sensor);
$n_{\mathrm{PM}}$—quantitative concentration of particles;
$\sigma_{\mathrm{scat}}(\lambda )$—scattering cross-section. It describes how much light a single particle scatters. It depends on the light wavelength λ and the particle size. The measurement algorithm is as follows: a laser penetrates the dust → the particle scatters light → a photodetector measures the scattering level → the PM count is obtained. General description of light attenuation in a medium:

(17) $$I(z)=I_{0}e^{-[\alpha_{\mathrm{gas}}(\nu )+\alpha_{\mathrm{PM}}(\lambda )]z}$$

where

$\alpha_{\mathrm{gas}}(\nu )$—molecular absorption, frequency-dependent;
$\alpha_{\mathrm{PM}}(\lambda )$—effective attenuation due to scattering and absorption of PM.

**Figure 3:**
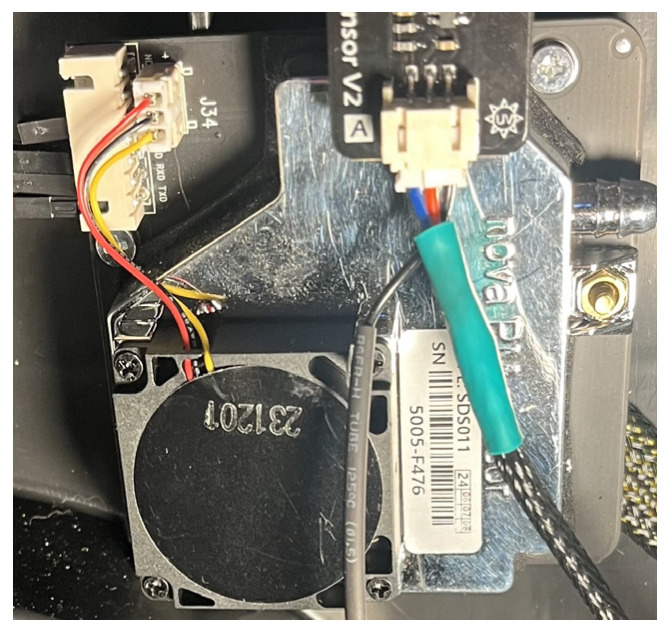
PM2.5 SDS011 sensor.

Both methods could be used separately or together. The intensity of the scattered light is proportional to the number of particles and their ability to scatter light. When a laser is shone through air containing PM [33], each particle scatters some of the light ($\sigma_{\mathrm{scat}}$). The more particles, the more scattering occurs, while the photo detector measures the total scattered light signal, determining the PM concentration. The measured detector signal is $I_{\mathrm{scattered}}$. Given the parameters of the optical system and $\sigma_{\mathrm{scat}}(\lambda )$, the inverse equation is solved; i.e., the quantitative concentration $n_{\mathrm{PM}}$ is obtained, then converted to mass concentration $(µg/m^{3}).$ The PM detection method was used in the construction of the remote measurement system (Figure 3).

A different method of gas detection is the electrochemical method [34,35,36] used for carbon monoxide gas detection. It uses compounds that change electrical resistance in the presence of the substance being detected. One such substance is tin dioxide, ${\mathrm{SnO}}_{2}$. In gas detectors, the ${\mathrm{SnO}}_{2}$ layer acts as an oxide semiconductor. In the case of ${\mathrm{CO}}_{2}$, the sensor does not react strongly, because ${\mathrm{CO}}_{2}$ is already an oxidized gas (it does not reduce oxygen on the ${\mathrm{SnO}}_{2}$ surface):

(18) $${SnO}_{2}+CO_{2}\to no\;reaction$$

When a reducing gas, e.g., carbon monoxide, is present, it reacts with oxygen and $O^{-}$ and ${O_{2}}^{-}$ ions are formed on the ${SnO}_{2}$ surface:

(19) $$CO+O^{-}\to CO_{2}+e^{-}$$

**Figure 4:**
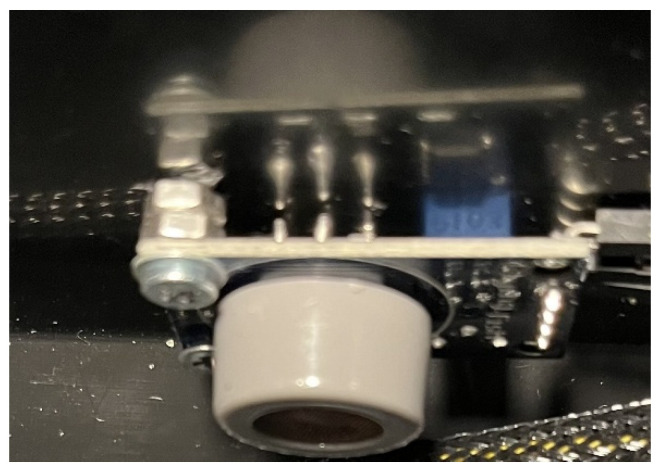
Semiconductor, MQ7, CO electrochemical sensor.

The released electrons reduce the sensor’s electrical resistance, which is the principle for gas detection. This type of sensor is used in the measurement module (Figure 4).

The carbon monoxide molecule absorbs infrared light [24], in the absorption bands of 4.6 and 2.3 µm. These absorption bands are used to detect gases. The weaker absorption band is 1600 nm, visible in spectroscopic studies, and 5 µm, detectable in high-sensitivity devices. Carbon monoxide does not absorb UV or visible light; it is transparent in these wavelengths. Carbon monoxide exhibits some absorption only at UV wavelengths below 15 µm. Sensors used in this method are divided into dispersive ones using prisms or gratings and non-dispersive ones using optical filters (NDIR technology). Other methods of carbon monoxide detection include

- Electrochemical detection, semiconductor-based;
- Chemiluminescent detection, which uses a reaction of compounds to emit light;
- Gas chromatography, which uses intermolecular interactions between the compounds being studied, and laser infrared spectroscopy, which uses a laser to measure absorption;
- Colorimetric detection, which changes the color of a substance in reaction with carbon monoxide.

**Figure 5:**
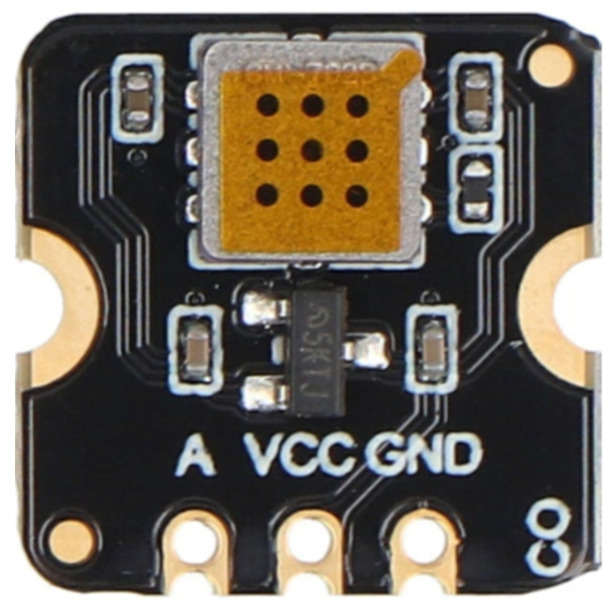
Carbon monoxide SEN0564 sensor.

An electrochemical carbon monoxide sensor was also used in this research (Figure 5).

Detection of hydrocarbons (HC) can be performed based on the absorption of infrared light [37]. The absorbed infrared wavelength is in the bandwidth of 3.3 to 3.5 µm. Due to the complexity and diversity of HCs, the wavelength of absorbed light (sodium hydroxide, potassium hydroxide) depends on their state of matter and chemical configuration. Hydrocarbons are transparent in the visible range but may exhibit ultraviolet absorption, most often in the range of about 200 to 300 nm. Detection of hydrocarbons can be performed using various chemical and analytical methods:

- Using pH indicators;
- Using the acid–base titration method, which involves the neutralization of ${OH}^{-}$ ions with acid;
- Using the reaction of hydrocarbons with metals, which produces hydrogen;
- Using the release of ammonia in the reaction with ammonium salts;
- Using the reaction of calcium hydroxide $\left({Ca(OH)}_{2}\right)$ with ammonia.

Nitrogen oxides $\left({NO}_{x}\right)$ absorb light primarily in the ultraviolet and visible ranges, and characteristic absorption bands may vary depending on their properties [38]. Nitrogen oxides exhibit absorption primarily in the infrared and ultraviolet ranges, mainly between 200 and 400 nm. Detection of nitrogen oxides can be performed by chemical analysis, using chemiluminescence. Nitrogen oxide reacts with ozone $\left(O_{3}\right)$, in the atmosphere, and the product of this reaction emits light. The intensity of this light is proportional to the NO concentration. The examples of nitrogen oxide detection methods include

- Spectroscopic, FTIR (Fourier Transform Infrared Spectroscopy) absorption, IR, UV;
- Methods based on electrochemical phenomena (yttrium-stabilized zirconium dioxide $ZrO_{2}$);
- Electrochemical, use of semiconductor sensors;
- Gas chromatography.

**Figure 6:**
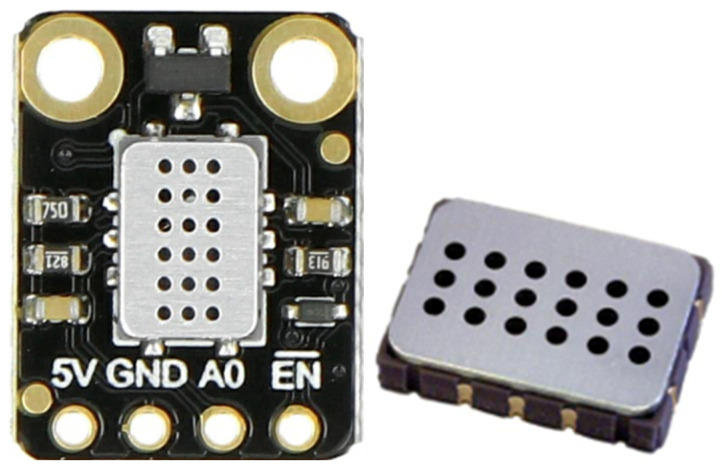
$NO/{NO}_{2}$ SEN0441 sensor.

For ${NO}_{x}$ detection, an electrochemical sensor was used in this research (Figure 6).

**Figure 7:**
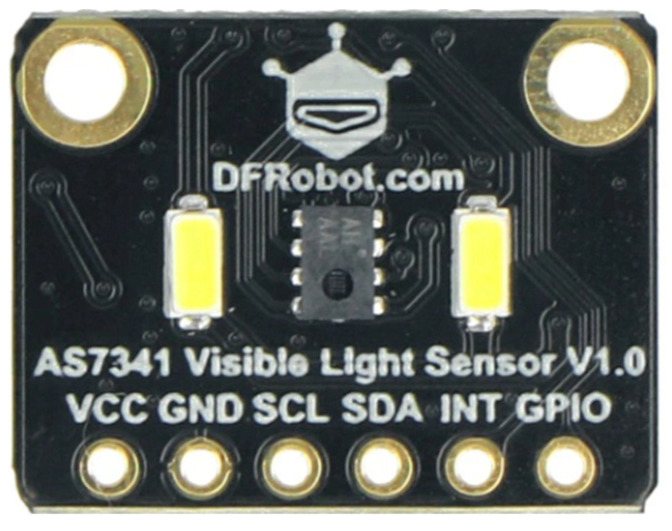
Visible light AS7341 sensor.

A visible light sensor was also used. It is an eleven-channel spectrometer with a spectral sensor (Figure 7).

**Figure 8:**
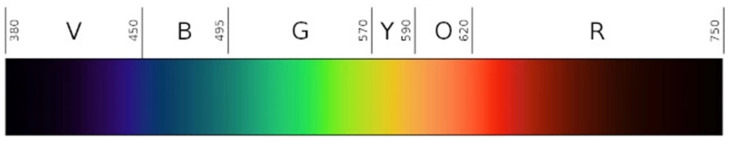
Visible light range.

**Figure 9:**
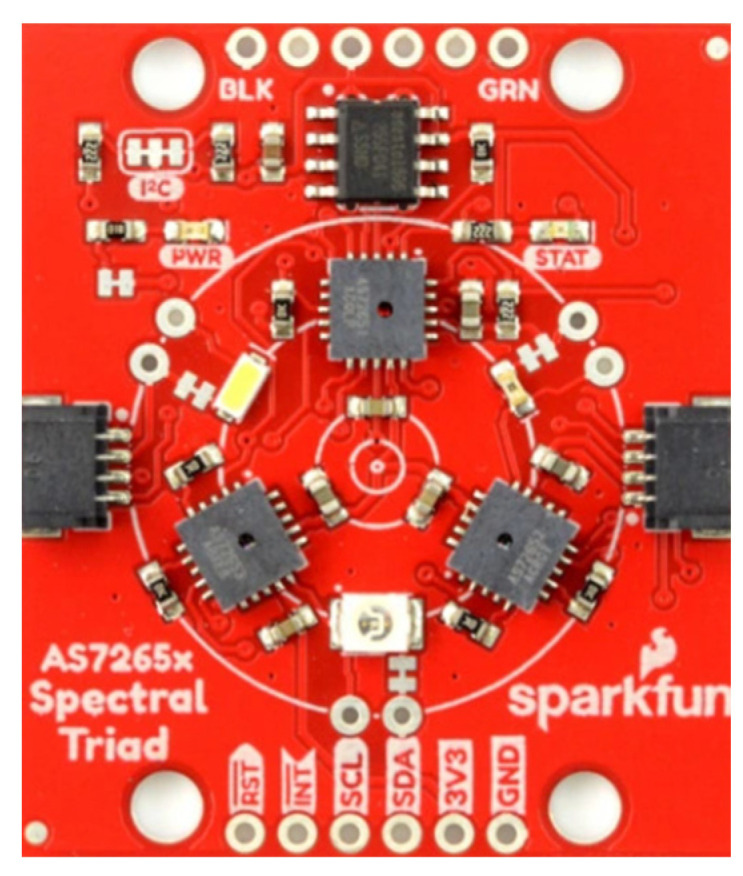
AS7265X spectrophotometer with 18 light frequency channels.

This sensor detects visible light and the near-infrared (IR) range (Figure 8). It identifies eight light channels from 400 to 690 nm (Figure 9), IR in the 840 to 1050 nm range, and a channel for flicker (lux). The spectrometer is illuminated by visible light, UV, laser, and infrared light.

The sensor’s response to detecting exhaust gas components is subject to examination, taking into account various electromagnetic wave sources. An 18-channel spectrophotometer, reading the UV to near-IR spectrum, with narrowband filters, was also used (Figure 9).

This broadband spectrophotometer analyzes spectral components in 18 visible and near-infrared light ranges. The sensor incorporates filters that are permeable to different wavelengths, allowing for a detailed image of the optical spectrum. The sensor detects smoke samples by identifying the reflected wave (using the sensor’s illumination) or by reading the light wave transmitted through the sample from an external source.

## 3. Description of the Detection System

**Figure 10:**
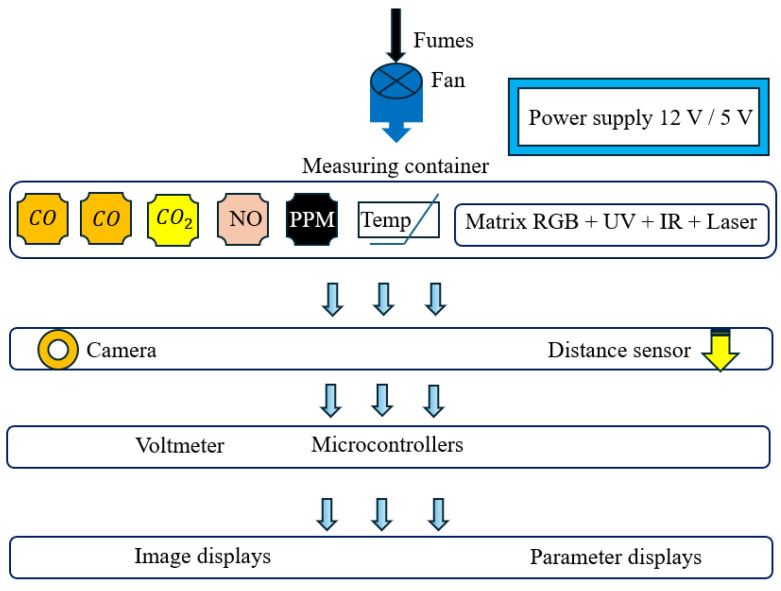
Schematic diagram of the system.

As was mentioned above, the sensory part of the system consists of a matrix of light elements that generate light of selected wavelengths, which are analyzed by spectrometers. The attenuation of these wavelengths in the contaminated gas is then determined. Each set consists of three diodes, including RGB-, laser-, ultraviolet-, and infrared-emitting diodes with different electromagnetic wavelengths, enhancing the system’s functionality. The light sources are used separately to prevent cross-influence of one light source on another. Using different detection media allows for drawing conclusions based on different data and combining the obtained information in a multi-criteria comparative analysis. The composition of the emission part of the “light sensor” is schematically shown in Figure 10 and described below:

- A set of three LEDs emitting ultraviolet light with wavelengths 260 to 270 nm, 365 nm and 395 to 405 nm, with a power of 1 to 3 W, powered by 12 V with 200 Ω resistors.
- RGB diodes emitting light wavelengths (blue: 450 to 495 nm; green: 495 to 570 nm; red: 620 to 750 nm), powered by 12 V. This diode emits total RGB light with the intensity set to maximum R, G, and B.
- LEDs in four colors with light wavelengths (blue: 450 to 495 nm; green: 495 to 570 nm; yellow: 550 to 650 nm; red: 620 to 750 nm), powered by 12 V with 470 Ω resistors.
- Three infrared diodes emitting light wavelengths 730 nm, 850 nm and 940 nm, with a power of 3 W, powered by 12 V with 220 Ω resistors.
- A 5 mW laser source with a wavelength of 650 nm, powered by 5 V.

All light sources were left unfiltered, meaning they were at their original light output, except for the laser source. Experiments revealed that the laser used filled the entire spectrometer reading spectrum. Leaving it at its factory output reduces the beam’s sensitivity to interference from identified air pollutants. This problem can be solved either by using optical solutions that extend the laser path to the beam reception point, or by appropriately selecting the power supply voltage. As a solution, attenuation of the laser beam with a filter was chosen, which increases the source’s sensitivity to smoke detection. A filter was experimentally selected to reduce the laser light intensity by approximately 80%. It was noted that during spectral reception using the light sources described above, additional wavelengths were visible, other than those specified by the diode manufacturers (Table 1). This increases the measurement capabilities of the constructed sensor, as detection involves comparing the undistorted wave with the smoke-distorted wave and determining the percentage difference. The subsystem described above is the basic one for the sensor set, while the CO and ${CO}_{2}$ sensors serve as auxiliary ones. Proper inference allows for estimating the probability that a given vehicle may be harmful to the environment in which it operates.

Light waves generated by the matrix of light elements were analyzed by the sensors placed in the measuring chamber. Table 1 below lists all the sensors used.

A physical challenge during mobile measurement is the temporary weather conditions, so a fan was used to increase the exhaust concentration in the measurement housing. However, the measurement carries the risk of obtaining unreliable data, so it should be repeated over a longer distance. The uncertainty of a mobile measurement, related to the vehicle moving before the measuring unit, is a “zero–one” definition, attempting to determine the probability of the object (car in front) exceeding or meeting the permissible standards.

The experimental vehicles were tested with a classic exhaust gas analyzer placed in the exhaust pipe, and the results served as the reference point for comparison with remote measurements. For obvious reasons (weather conditions, wind, etc.) and because of exhaust gases’ dispersion in the air, remote measurements cannot accurately relate the results to measurements taken directly in the vehicle’s exhaust pipe. This is typical in the remote gas measurement and was also discussed in the papers [10,13,14,16,17], referenced in the Introduction chapter. In the remote method described in this paper, only the possibility of the tested vehicle failing to meet the exhaust gas standards can be determined.

## 4. Results

The results of tests using the constructed sensory module are presented below. The measurements were performed remotely, meaning that the exhaust gases were drawn into the measuring housing while maintaining a certain distance between the exhaust pipe of the vehicle under test and the inlet of the remote system. Basically, the degree of response depends on the distance between the measuring system and the tested vehicle. It should also be noted that the measurements were taken while the engines were idling. If the discussed sensor system were to be implemented for on-road measurements, the results would be more significant due to the larger volume of generated exhaust gases (increased engine load) and higher levels of toxic and undesirable substances.

The measurement methodology involves determining the reduction in electromagnetic wave intensity $\left({∆}_{I_{smoke}}\right)$, caused by the wave passing through polluted air, compared to the original measurement $I_{0}$. This change is due to absorption at specific wavelengths, but in the ranges used, this phenomenon can be described as “weak” absorption, extending beyond the absorption bands characteristic of the given elements. An additional phenomenon is light scattering in various directions, resulting in a reduction in wave amplitude and lower light intensity reaching the spectrometer detector. As a result, the difference $\left({∆}_{I_{smoke}}\right)$, which serves as a measurement parameter, was obtained. Physically, this is represented by Equation (4). In this equation, the parameter *α*, defining the absorption coefficient, actually consists of two components: the absorption and scattering of the wave (20).

(20) $$\alpha =\alpha_{absor+disper}=\alpha_{absor}+\alpha_{disper}$$

As a result, the light intensity obtained from the measurements has the dimension of Equation (4), where $\alpha =\alpha_{absor+disper}$. Table 1 and Table 2 present the test results for vehicles with compression ignition and spark ignition engines, determining the attenuation of the intensity of the electromagnetic wave $\left({∆}_{I_{smoke}}\right)$, described as a percentage.

Vehicles exceeding the permissible exhaust emission standards for carbon monoxide and PMs were selected for the measurements. The measurements were repeated several times, resulting in signatures (light wavelengths) indicating the probability that the vehicle should be subjected to more thorough verification in stationary measurements. Results below 10% $\left({∆}_{I_{smoke}}\right)$ were omitted. Below are the signatures, composed of the electromagnetic wavelengths at which $\left({∆}_{I_{smoke}}\right)$ was defined, characteristic for the type of fuel used by the vehicle, selected from Table 2 and Table 3.

- Compression ignition (CI):
  ○ IR: 505 to 545 nm and 580 to 600 nm.
  ○ Visible light: 620 to 640 nm, 780 to 940 nm.
  ○ Laser: 435 to 455 nm, 620 to 640 nm, 670 to 690 nm, 780 to 940 nm.
- Spark ignition (SI):
  ○ IR: 505 to 545 nm, 580 to 600 nm.
  ○ Visible light: A reduction in light intensity was observed throughout the entire wavelength range.
  ○ Laser: 405 to 425 nm, 470 to 490 nm, 780 to 940 nm.

The results from the spectrometers were combined with the readings from the CO, ${CO}_{2}$, and PM sensors. Table 4 presents the air pollution index according to European Environment Agency [39].

In the further considerations, references to the ppm units were omitted, while the presented results refer comparatively to Table 3.

The purpose of a diesel particulate filter (DPF) is to capture soot particles. The amount of these particles measured at the exit of the vehicle’s exhaust system indicates the DPF’s efficiency in performing this function. When selecting system components, numerous experiments were conducted, which determined the dispersion of PM measurements for various vehicles. The measured amount of PM passing through the DPF is not a fixed quantity. It depends on the current operating conditions, engine type, its technical condition, the condition of the engine management system, fuel quality, and type of operation. However, this quantity varies slightly for a given vehicle in subsequent measurements. Therefore, PM ranges corresponding to specific states are provided, determining the efficiency or presence of a DPF in the vehicle.

The PM level readings for vehicles equipped with a diesel particulate filter (DPF) were within the following ranges:

$$\begin{array}{c} {PM}_{2.5}:\;20÷120\\ {PM}_{10}:\;20÷120 \end{array}$$

The result depends on the background level, or air quality at the location where the test was conducted. The given values correspond to the actual air pollution at the place of measurement (between “good” and “very poor” in Table 4). Taking that into account allows for a conclusion that this level for the vehicles in proper condition falls within the following ranges:

$$\begin{array}{c} {PM}_{2.5}:\;8÷180\\ {PM}_{10}:\;8÷180 \end{array}$$

It is possible that the background may contain more PMs than the exhaust from a vehicle equipped with a diesel particulate filter (DPF). Comparing the measurement results with the limits shown above, the detection of whether the vehicle’s DPF is functioning is possible. The excess is so significant that it leaves no doubt as to the condition of the DPF. If the DPF is not working properly or is missing, the PM level can differ by up to twenty times compared to a properly functioning vehicle. In tests of cars without DPF, the result obtained is a spread between

$$\begin{array}{c} {PM}_{2.5}:\;600÷2000\\ {PM}_{10}:\;600÷2000 \end{array}$$

**Figure 11:**
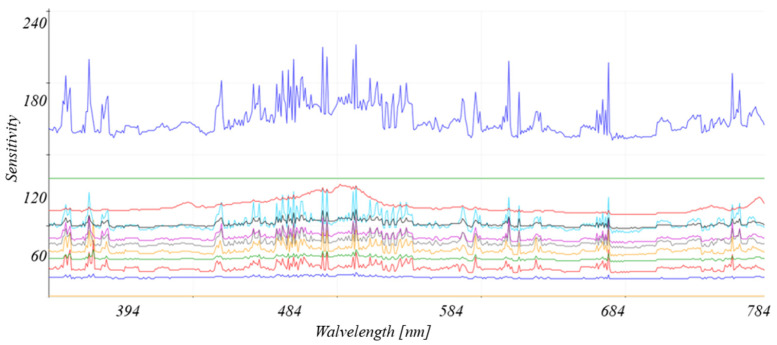
Visualization of the obtained light levels for each identified wavelength of visible light.

This significant difference between vehicles with and without a DPF allows for a definite determination of whether the vehicle in question has a properly working DPF or not. The identified CO and $CO_{2}$ levels did not demonstrate significant differences in the detection of these gases, but still they are worth noticing, leading to the conclusion that, in most cases, permissible standards are likely exceeded. In the case of hydrocarbons, their presence is visible in the lower ultraviolet range. In the spectroscopic measurements, the range of identified electromagnetic wavelengths was extended from 200 to 370 nm by using an additional optoelectric sensor based on a gallium nitride semiconductor. Figure 11 shows an example reading of the levels of identified wavelengths read by the spectroscope.

The spark ignition vehicle, meeting the EURO 3 standard, exceeded the CO standard (2.3 g/km) twice. The tests revealed weak absorption bands at 440 to 550 nm within the range of identified electromagnetic wavelengths, as well as a 25% reduction in wavelength source detection associated with the high CO parameter detected by the sensor using the photoacoustic effect. At the same time, a significant increase in particulate matter was detected, reaching values of 998 ${PM}_{2.5}$ and 1982 ${PM}_{10}$, as determined by laser detection. The CO sensor indicated a two-fold increase in this parameter compared to the clean air measurement (from 121 to 265, the unidimensional value). Testing a diesel vehicle (EURO 4, CO up to 0.5 g/km), whose emissions exceeded the permissible limits by more than two times, lead to the following results: a weak electromagnetic absorption band was identified at 620 nm to 690 nm, as well as a 20% reduction in source identification and an increase in ${CO}_{2}$, CO, and PM parameters to values of 587 for ${PM}_{2.5}$ and 688 for ${PM}_{10}$. This vehicle did not have a DPF. If the measurement had been performed directly at the exhaust pipe, the PM measurement would have been higher, but the value determined in the measurements is sufficient to confirm the absence of a particulate filter in the vehicle. Spectrometric readings are associated with high PM levels and are complementary to the measurements, as are readings from carbon monoxide and carbon dioxide sensors. Additionally, tests were conducted using only carbon monoxide and carbon dioxide (not derived from vehicle exhaust) drawn into the measurement container (Figure 10). The results are presented in Table 5 and Table 6.

The results in Table 4, describing carbon monoxide measurements, refer to the results of a spark ignition vehicle (SI). The results in Table 5, describing carbon dioxide measurements, are to be applied to a compression ignition vehicle (CI). This is because the primary pollutant in CI is soot (PM), while in spark-ignition (SI) vehicles, it is carbon monoxide. Obviously, there are gasoline-powered vehicles that generate high PM readings due to engine wear. Carbon monoxide signature (SI engine) is as follows:

○ IR: 435 to 600 nm, 580 to 600 nm, 670 to 940 nm;
○ Visible light: 435 to 405 nm, 620 to 690 nm;
○ UV: 405 to 425 nm;
○ Laser: 435 to 490 nm, 620 to 940 nm.

Carbon dioxide and carbon monoxide signature (CI engine):

○ IR: 505 to 545 nm, 580 to 600 nm, 670 to 690 nm;
○ Visible light: 620 to 640 nm;
○ Laser: 435 to 490 nm, 620 to 940 nm.

The results of ${∆}_{I_{smoke}}$, i.e., the reduction in intensity, are consistent with the exhaust gas tests in the exhaust pipe. Due to the concentration of the injected gas, additional characteristic points appear for CO and ${CO}_{2}$. The differences concern the use of visible light, in which range the reaction for CO and ${CO}_{2}$ is point-like, and the exhaust gas generated by a gasoline vehicle distorts the entire light spectrum. The presented signatures are threshold values defining the exceedance of permissible levels of undesirable substances. Several measurements were taken to confirm the obtained results. It should be noted that “remote” measurements are subject to uncertainty resulting from the measurement method itself. There was no measuring probe mounted in the vehicle’s exhaust pipe, so the measurement result depended on external conditions. Therefore, a series of measurements was performed. Hence, the term “probability of exceeding exhaust gas standards” was used, not “certainty.” In the conducted experiments, it was observed that vehicles with normal exhaust gas emissions were not visible to the sensors. Their exhaust gases do not interfere with the light intensity of the sources used.

## 5. Summary

In this paper, the possibility of creation of the relatively inexpensive diagnostic system has been discussed, which could be implemented on a patrol vehicle enabling remote measurements of exhaust emissions from automotive combustion engines during their normal road operation. The system’s purpose is to determine the likelihood of a given vehicle’s potential harmfulness to the environment. The sensor system utilizes electrochemical, optoelectrical, and laser-based sensors. A light array composed of visible, ultraviolet, and infrared light sources was incorporated, working in conjunction with spectrometers. The light is received by spectrometer detectors, which transmit information about light detection levels for various electromagnetic wavelengths. The physical phenomena used include absorption, light scattering, and photoacoustic and electrochemical phenomena. The kit has been enhanced with proprietary solutions that extend its functionality. The system is managed by dedicated software, and multi-criteria parameter analysis allows for accurate conclusions based on the conducted tests. The sensor system was built as a mobile laboratory, allowing for the performance of various measurement experiments.

This system, implemented in a patrol vehicle, could detect vehicles posing a threat to the environment on the road. Installed in the front of a roadside measurement vehicle, it could conduct numerous roadside inspections, leading to the identification of potential environmental hazards.

## Figures and Tables

**Table 1:** List of sensors used in the system.

Sensor	Producer	Type of Sensor
${CO}_{2}$	Infineon Technologies AG	XENSIV PASC02V1
PM	Nova Fitness Co., Ltd.	PM2.5 SDS011
CO	Henan Hanwei Electronics Co., Ltd.	MQ7
CO	SEN0564	SEN0564
NO, NO_2_	DFRobot	SEN0441
9 ch. spectroscope	DFRobot	AS7341
18 ch. spectrometer	Toshiba Electronic Devices	AS7265X

**Table 2:** Electromagnetic wave attenuation, vehicle CI.

		Compression Ignition			
Length [nm]	3 IR	4 DI	3UV	RGB	Laser
405 ÷ 425	92	100	97	100	100
435 ÷ 455	100	96	100	97	78
470 ÷ 490	100	100	100	96	96
505 ÷ 545	71	100	100	98	100
545 ÷ 565	100	88	100	100	100
580 ÷ 600	85	94	100	96	188
620 ÷ 640	100	72	92	78	79
670 ÷ 690	100	100	100	100	82
780 ÷ 940	100	43	100	58	76
Lx	100	82	100	97	100

**Table 3:** Electromagnetic wave attenuation, vehicle SI.

		Spark Ignition			
Length [nm]	3 IR	4 DI	3UV	RGB	Laser
405 ÷ 425	100	100	100	67	70
435 ÷ 455	100	44	95	59	94
470 ÷ 490	100	100	100	59	64
505 ÷ 545	71	100	95	57	100
545 ÷ 565	100	100	88	64	100
580 ÷ 600	89	100	100	25	100
620 ÷ 640	100	62	92	76	100
670 ÷ 690	100	100	100	67	91
780 ÷ 940	100	100	100	80	57
Lx	100	86	88	60	56

**Table 4:** EEA air pollution levels.

Pollution			Index	Level [ppm]		
	Good	Fair	Moderate	Poor	Very Poor	Extremely Poor
${PM}_{2.5}$	0÷10	10÷20	20÷25	25÷50	50÷75	75÷800
${PM}_{10}$	0÷20	20÷40	40÷50	50÷100	100÷150	150÷1200

**Table 5:** Attenuation of the electromagnetic wave intensity (CO) [%].

		Carbon Monoxide			
Wavelength [nm]	IR	DI	3UV	RGB	Laser
405 ÷ 425	100	100	46	100	100
435 ÷ 455	76	22	100	94	69
470 ÷ 490	67	23	100	92	92
505 ÷ 545	71	100	100	98	100
545 ÷ 565	88	100	100	100	100
580 ÷ 600	33	100	100	100	100
620 ÷ 640	100	82	100	94	69
670 ÷ 690	82	100	67	100	73
780 ÷ 940	94	100	100	100	59
Lx	100	100	69	94	49

**Table 6:** Attenuation of the electromagnetic wave intensity (${CO}_{2}$) [%].

Carbon Dioxide					
Wavelength [nm]	IR	DI	3UV	RGB	Laser
405 ÷ 425	92	100	95	100	100
435 ÷ 455	100	97	100	93	91
470 ÷ 490	100	95	91	92	80
505 ÷ 545	71	100	100	98	100
545 ÷ 565	100	100	88	100	100
580 ÷ 600	78	100	90	100	100
620 ÷ 640	100	84	92	96	100
670 ÷ 690	49	100	100	100	82
780 ÷ 940	100	73	100	100	61
Lx	100	65	98	94	60

## Data Availability

The original contributions presented in this study are included in the article. Further inquiries can be directed to the corresponding author.
